# Aroma enhancement in fig wine through sequential fermentation with *Candida humilis* and *Torulaspora delbrueckii*: A flavoromics study

**DOI:** 10.1016/j.fochx.2026.103855

**Published:** 2026-04-09

**Authors:** Bing Han, Jian Ma, Shuai Li, Haihong Wu, Yu Wang, Xingye Wang, Yanhong Ma

**Affiliations:** a Institute of Agro-Product Processing, Jiangsu Academy of Agricultural Sciences, Nanjing, 210014, China; b Key Laboratory of Green and Low-carbon Processing Technology for Plant-based Food of China National Light Industry Council, School of Food and Health, Beijing Technology and Business University, Beijing 100048, China; c Shanxi Center of Technology Innovation for Storage and Processing of Fruit and Vegetable, Shanxi Agricultural University, Taigu, 030801, China; d Xingjiang Haidekun Agricultural Technology Co., Ltd., Artush 845350, China

**Keywords:** *Β*-Glucosidase, Esterase, Indigenous yeasts, Volatile compounds, Fig wine

## Abstract

Three yeast strains isolated from fig fruits (C*andida humilis* DBXD1, *Torulaspora delbrueckii* S14, and *Saccharomyces cerevisiae* S2–7) were evaluated for their potential to enhance the aromatic complexity of fig wines. Enzyme activity assays demonstrated that *C. humilis* DBXD1 exhibited maximum *β*-glucosidase activity (2.94 ± 0.13 U/mg prot) and *T. delbrueckii* S14 showed the highest esterase activity (0.94 ± 0.06 U/mg prot). Sequential inoculation of these non-*Saccharomyces* strains with *S. cerevisiae* S2–7 increased contents of key aroma compounds in fig wines, including ethyl acetate, isoamyl acetate, hexyl acetate, butanoic acid esters, isoamyl alcohol, nonanal, 2-nonanone, and α-terpineol. Metabolic pathway analysis identified valine, leucine and isoleucine degradation, glycine and serine metabolism, and the glucose-alanine cycle as the primary differential pathways contributing to flavor enhancement. These findings demonstrated that sequential inoculation with indigenous high enzyme-producing yeasts provides a practical foundation for industrial production of fig wines with distinctive aromatic characteristics.

## Highlights

• The fig wines were fermented with three high enzyme-producing yeasts.
• Sequential fermentation improved polyphenols and antioxidant activity of fig wines.
• Sequential fermentation increased esters and higher alcohols of fig wines.
• DBXD1 and S14 co-inoculation enhanced floral and fruity aromas of fig wines.
• Valine, leucine and isoleucine degradation was the key pathway to enhance flavor.

## 1 Introduction

Fig (*Ficus carica* L.), an important berry fruit belonging to the Moraceae family, is widely cultivated in Xinjiang, Shandong, and Sichuan provinces of China (Ma et al., 2022). Due to its sweet taste, unique flavor, and rich nutrients (polyphenols, flavonoids, amino acids, and vitamins), fig is highly favored by consumers (Salma et al., 2020; Solomon et al., 2006). However, figs are highly perishable with a short postharvest life due to their thin skin, high respiration rate, and susceptibility to microbial spoilage during hot seasons, which rapidly deteriorates their color, texture, flavor, and nutritional value, thereby compromising their edible quality and economic viability (Ma et al., 2022). Thus, further research on converting figs into long-lasting and high-value products has attracted great attention (Teruel-Andreu et al., 2021). Compared with non-fermented products such as dried fruits and jams, the processing of fig wine can preserve the unique flavor and biological activity of figs, which presents a sustainable solution to minimize wastage and generate additional financial returns (Teruel-Andreu et al., 2021). Despite the great benefits, severe flavor homogeneity and lack of desired aroma complexity are imminent challenge facing fig wine products.

The aromatic characteristics of fruit wines are fundamentally determined by the metabolic activities of yeasts. While spontaneous fermentation can impart regional typicity to fruit wines (Chen et al., 2023), its inherent unpredictability, risk of stuck fermentation, and prolonged fermentation period limit its industrial applicability. Inspired by the microbial diversity of spontaneous fermentation, the strategic application of mixed starter cultures comprising indigenous *Saccharomyces cerevisiae* and non-*Saccharomyces* yeasts has emerged as a promising approach to enhance wine aroma complexity while maintaining process control (Liu et al., 2020; Zhang et al., 2022). Non-*Saccharomyces* yeasts contribute to aroma enhancement primarily through their enzymatic activities. These yeasts secrete *β*-glucosidase, which hydrolyzes glycosidically-bound aroma precursors present in fruits, releasing volatile aglycones that contribute to floral and fruity notes (Liu et al., 2022). Additionally, they produce esterase involved in the synthesis and hydrolysis of short, medium, and long-chain aliphatic esters, directly modulating the ester profile of wines (Sun, Chen, Bi, et al., 2024). Numerous studies have documented the positive contributions of specific non-*Saccharomyces* yeasts. For instance, *Torulaspora delbrueckii*, *Lachancea thermotolerans*, and *Metschnikowia pulcherrima*, have been shown to impart fruity and floral aromas to wines via influencing ester biosynthesis (Renault et al., 2015). In Gewürztraminer wine, inoculation with *β*-glucosidase-producing *Candida glabrata* intensified floral and fruity aroma attributes (Han et al., 2021). Similarly, high enzyme-producing strains including *Zygosaccharomyces rouxii*, *Meyerozyma guilliermondii*, and *Pichia kudriavzevii* have been observed to increase the content of ethyl esters, acetates, and higher alcohols, enhancing the floral and tropical fruit aromas of kiwifruit wine (Sun, Chen, Feng, et al., 2024). Despite their promising enzymatic and aromatic production abilities, it should not be ignored that non-*Saccharomyces* yeasts cannot independently be responsible for sugar-ethanol conversion due to their poor tolerant to oenological conditions (Han et al., 2021). *S. cerevisiae* remains the dominant species for alcoholic fermentation because of its robust sugar-ethanol conversion ability and strong stress tolerance (Chen et al., 2022). However, it was suggested that *S. cerevisiae* showed weaker *β*-glucosidase activity compared to non-*Saccharomyces* yeasts, especially under fermentation conditions characterized by high sugar, alcohol, and sulfur content (López et al., 2015; Wang, Fan, et al., 2023). This functional complementarity has motivated the development of sequential fermentation strategies, wherein non-*Saccharomyces* yeasts are inoculated first to exert their enzymatic effects, followed by *S. cerevisiae* to ensure complete and efficient alcoholic fermentation (Gao et al., 2022; Shi et al., 2019).

The application of sequential fermentation to enhance fruit wine quality has gained considerable research attention. Previous studies have suggested that sequential inoculation with *T. delbrueckii* and *S. cerevisiae* increased the content of ethyl 3-methylbutanoate, ethyl hexanoate, and ethyl octanoate, imparting fruity and sweet notes to blueberry wine (Wang, Qi, et al., 2023). Sequential fermentations employing *Schizosaccharomyces pombe* and *S. cerevisiae* not only increased polyphenol content, but also enhanced floral and sweet notes in kiwifruit wine (Li et al., 2022). However, the outcomes of sequential fermentation are highly dependent on the specific yeast strains employed, the physicochemical characteristics of the fruit substrate, and the complex microbial interactions that occur during co-inoculation (Muñoz-Redondo et al., 2021). Therefore, strain selection and process optimization must be tailored to each specific fruit matrix.

To date, the enzymatic potential of indigenous fig-associated yeasts remains largely unexplored, and the impact of sequential inoculation on fig wine aroma profile has not been systematically characterized. Given the unique biochemical composition of figs, there is significant potential for non-*Saccharomyces* yeasts to unlock fig-specific aromatic complexity through their enzymatic activities. Therefore, the present study aimed to characterize the *β*-glucosidase and esterase activities of three indigenous yeasts isolated from fig fruits, comprehensively profile the amino acids, polyphenols, and volatile compounds produced under different fermentation regimes, and establish correlations between fermentation strategies and the resulting aromatic profiles. Overall, the findings of this work will provide a scientific basis for developing optimized starter cultures tailored specifically for fig wine production, contributing to the diversification and quality improvement of fig-based products.

## 2 Materials and methods

### 2.1 Yeast strains

*Candida humilis* DBXD1 (CGMCC No. 32666), *Torulaspora delbrueckii* S14, and *Saccharomyces cerevisiae* S2–7 (CGMCC No. 33390) were previous isolated in our laboratory from the spontaneous fermentation of fig fruits (Halajun, Xinjiang, China), and identified by ITS sequencing as described by Han et al. (2021). Those strains have been proven to possess high *β*-glucosidase and esterase activity and kept frozen at −80 °C in 25% glycerol (1:1, *v*/v).

### 2.2 Characterization of β-glucosidase and esterase activity of yeast strains

#### 2.2.1 Extraction, purification, and quantification of β-glucosidase and esterase of yeast strains

Referring to the method of Sun, Chen, Bi, et al. (2024), the strains were inoculated in an enzyme-producing medium (Yeast extract 10 g/L, peptone 20 g/L, glucose 20 g/L, (NH_4_)_2_SO_4_ 3 g/L, KH_2_PO_4_ 4 g/L, MgSO_4_·7H_2_O 0.5 g/L, Tween 80 10 mL/L) at 28 °C for 72 h. After centrifugation (10,000 ×*g*, 15 min, 4 °C), the cell-free supernatant was mixed with ammonium sulfate until a saturation of 70% was achieved, followed by standing at 4 °C for 24 h. The resulting solution was centrifuged (10,000 ×*g*, 15 min, 4 °C) and the precipitate was dissolved in an acetic acid‑sodium acetate buffer (0.1 M, pH 4.5). Then, the supernatant obtained by repeated centrifugation (10,000 ×*g*, 15 min, 4 °C) was transferred to a 14 kDa dialysis bag at 4 °C for 24 h. The distilled water was used as dialysis solution. Finally, the purified enzyme solution was stored at 4 °C for activity assay.

The activities of *β*-glucosidase and esterase in extracellular extracts of yeast strains were quantified using the 4-nitrophenyl *β*-D-glucopyranoside (p-NPG) method and the 4-nitrophenyl butyrate (p-NPB) method, respectively. For *β*-glucosidase activity, 0.2 mL of the purified enzyme solution was mixed with 0.2 mL p-NPG (35 mM) and 1.5 mL of citric acid phosphate buffer (0.1 M, pH 5.0). After keeping in a thermostatic water bath at 50 °C for 30 min, 2 mL of sodium carbonate (1 M) was added to terminate the reaction. For esterase activity, 0.2 mL of purified enzyme solution was mixed with 40 μL of p-NPB (25 mM) and 0.76 mL of citric acid phosphate buffer (0.1 M, pH 5.0). Then, the reaction solution was inoculated at 40 °C for 60 min, and 100 μL of sodium hydroxide (0.5 M) was added to stop the reaction. The absorbance value of the reaction solution at 400 nm was measured using the heat-inactivated enzyme solution as a control. All enzyme activity assays were performed in triplicate (three independent biological replicates for each yeast strain, with each biological replicate assayed in technical duplicate). The activity unit (U) was defined as the amount of enzyme required to catalyze the release of 1 μmol of p-nitrophenol (p-NP) within 1 min. The concentration of p-NP was calculated based on the standard curve, which was established via the linear regression model relating the absorption at 400 nm and the corresponding p-nitrophenol concentration (10, 20, 40, 50, 80, 100 μmol/L). The protein content was determined following the instruction of the total protein assay kit (Nanjing Jiancheng, Nanjing, China). The specific activity of the enzyme was calculated as follows:

Specific activity (U/mg prot) = enzyme activity (U/mL) / protein content (mg/mL).

#### 2.2.2 The stress tolerance of yeast strains to the oenological conditions

Five parameters that are crucial to fig wine fermentation were taken into account: temperature, pH value, glucose, ethanol, and sulfur dioxide. To clarify the stress tolerance of yeast strains to the oenological environment, an initial cell density of 6 log CFU/mL were inoculated in YPD broth (Aobox, China). Afterward, the culture was cultured at 10, 15, 20, 25, or 30 °C for 72 h, or adjusted to a certain range of pH values (3, 4, 5, 6, or 7) and incubated at 25 °C for 72 h. To analyze the effects of fig wine matrix on growth activity of those strains, glucose (100, 150, 200, 250, or 300 g/L), ethanol (0, 4, 8, 12, or 16%(*v*/v)), and sulfur dioxide (0, 30, 60, 90, or 120 mg/L) with different final concentrations were added to the medium, respectively, and the strains were incubated at 25 °C for 72 h. The 600 nm absorbance of the finial culture were measured by UV spectrophotometer (Shimadzu UV-1800, Japan).

### 2.3 Fig wine fermentation

Referring to the method of Ma et al. (2022), the fig fruits harvested in July 2025 from the Cold and Cool Forest Fruit Industrial Park (Halajun, Xinjiang, China) were squeezed using a juicer (Mr. Orange, JP-2, China). Pectinase (0.3%) was added for enzymatic digestion at 55 °C for 2.5 h and the apricot must was centrifuged (10,000 ×*g*, 15 min, 4 °C) to obtain clarified juice. A total of 2.0 L clarified juice was sterilized by filtrations using first 0.65 μm and then 0.45 μm filter film in each 2.5 L fermentation bottle, 3 parallels were applied for each fermentation. Then, potassium bisulfite (K_2_S_2_O_5_) was added to obtain free sulfur dioxide at 30 mg/mL and to inhibit the growth of bacteria during the fig wine fermentation process (Lu et al., 2021; Ma et al., 2022). Afterwards, the sugar content was adjusted to 23°Brix, and the fig juice was ready to inoculate yeast strains. The inoculation schemes were as follows: (1) the commercial yeast *S. cerevisiae* DV10 (Lallemand, Canada) pure culture; (2) *S. cerevisiae* S2–7 pure culture; (3) *C. humilis* DBXD1 was 2 d prior to *S. cerevisiae* S2–7; (4) *T. delbrueckii* S14 was 2 d prior to *S. cerevisiae* S2–7; (5) *C. humilis* DBXD1 and *T. delbrueckii* S14 (1:1) was co-inoculated 2 d prior to *S. cerevisiae* S2–7. As a positive control, the commercial yeast *S. cerevisiae* DV10 was rehydrated in hot water (37 °C) and added into bottles with a concentration of 0.2 g/L. These high enzyme-producing yeast strains were pre-incubated in YPD medium at 28 °C for 24 h, and the initial inoculum was 6 log CFU/mL. The fermentations were carried out at 20 °C and sampled at days 0, 2, 4, 6, 8, 10, 12, and 16. At each time point, approximately 40 mL of fermenting must was withdrawn from each fermentation vessel under sterile conditions. The sample was immediately divided into aliquots for different analysis. All samples were processed within 30 min of collection to minimize metabolic changes. Frozen samples were analyzed within one month of collection.

Yeast colony number was detected using the colony plate counting method, °Brix and pH value were detected by handheld portable devices, total acidity was measured using the neutralization titration method, and the chromatic properties were characterized using a CS-820 colorimeter (Caipu Technology Co., Hangzhou, China). When the alcoholic fermentation finished (the residual sugar stabilized below 4.0 g/L), the fig wines were centrifuged (10,000 ×*g*, 15 min, 4 °C) and the supernatant was collected. The volatile compound determination, sensory analysis, electronic nose (*E*-nose) and electronic tongue (E-tongue) analysis were conducted within 48 h of fermentation completion to minimize storage-related changes. The remaining were kept at −80 °C for chromatographic analysis of sugars, glycerol, ethanol, organic acids, free amino acids, and phenolic compounds. Each aliquot was thawed only once immediately before analysis to avoid freeze-thaw cycles. All analytical measurements were performed in technical duplicate to account for measurement variability.

### 2.4 Determination of sugars, glycerol, ethanol, and organic acids

Based on our previous published method (Han et al., 2025), the glucose, fructose, glycerol, and ethanol were quantified using an HPLC system equipped with a refractive index detector (Waters, MA, USA). Chromatographic separation was performed on a Aminex HPX-87H column (300 × 7.8 mm, 9 μm) with a column temperature of 55 °C and a flow rate of 0.5 mL/min. Organic acids were determined by the HPLC system coupled with a diode array detector (Waters, MA, USA). Chromatographic separation was conducted with the same column at 40 °C and 0.6 mL/min flow rate. The mobile phase was 5 mM sulfuric acid, the detection wavelength was 210 nm, and the injection volume was 10 μL. Qualification referred to retention time and quantification was calculated based on the corresponding calibration curve of standards.

### 2.5 Determination of free amino acids

Referring to the method of Fontes et al. (2024), the free amino acids were detected by pre-column derivatization and chromatographic analysis. Firstly, 50 μL of the sample was mixed with 150 μL *Orto*-phthalaldehyde (10 mg/mL) and 300 μL borate buffer (40 mM, pH 9.5) for 15 min. Then, 25 μL of the derivatized sample was injected into the HPLC system (Waters Corp., MA, USA) equipped with an Agilent Zorbax Eclipse Plus C18 column (100 × 4.6 mm, 3.5 μm). The column temperature was 40 °C, the flow rate was 1.5 mL/min, and the detection wavelength was 338 nm. The gradient elution procedure composed of (A) acetonitrile-methanol-water solution (in a 45:45:10 vol ratio) and (B) 10 mM sodium phosphate buffer-10 mM sodium borate (pH 8.2) was as follows: 0–0.35 min 2% A, 0.35–13.4 min 2–57% A, 13.4–13.5 min 57–100% A, 13.5–16.5 min 100% A, 16.5–17 min 100–2% A, 17–18.5 min 2% A. Calibration curve was established via the linear regression model relating the peak area of each amino acid and the corresponding concentration.

### 2.6 Determination of total phenol, total flavonoid, and phenolic compounds

The total phenol content was determined by the Folin-Ciocalteu method (Ma et al., 2022). Briefly, 0.2 mL of the diluted sample was mixed with 0.5 mL of Folin-Ciocalteu reagent, 2 mL of 7.5% sodium carbonate solution, and 2.3 mL of deionized water. Then, the mixture was incubated at 20 °C for 2 h, and the absorbance value at 760 nm was measured. The standard curve of gallic acid was constructed as the above method, and the results were expressed as gallic acid equivalents.

The total flavonoid content was determined by the aluminum nitrate method (Pu et al., 2023). In brief, 1 mL of the diluted sample was mixed with 0.5 mL of 5% sodium nitrite, 0.5 mL of 10% aluminum nitrate, 4 mL of 4% sodium hydroxide, and 4 mL of deionized water. Then, the mixture was shaken for 5 min and measured at an absorbance of 510 nm. The standard curve of rutin was established as the same method, and the results were expressed as rutin equivalents.

According to the method of Anjos et al. (2024), the phenolic compounds were detected by ethyl acetate extraction and chromatographic analysis. Briefly, 10 mL of the sample was mixed with 20 mL of ethyl acetate and stirred in the vortex for 15 min. After centrifugation (10,000 ×*g*, 10 min, 4 °C), the upper organic phase was transferred to another tube, and 20 mL of ethyl acetate was added for the second extraction. Then, the merged extraction solution was rotary evaporation and the dried residue was dissolved in methanol before being separated by the HPLC system (Waters Corp., MA, USA). Chromatographic separation was performed on a Waters xTerra MS C18 column (250 mm × 4.6 mm, 5 μm) with a 1.0 mL/min flow rate. The column temperature was 30 °C, the injection volume was 20 μL, and full wavelength scanning was performed. The gradient elution program composed of (A) methanol and (B) 1% acetic acid aqueous solution was as follows: 0–10 min 5–30% A, 10–15 min 30–50% A, 15–25 min 50–70% A, 25–30 min 70–5% A. The identification and quantification of phenolic compounds were accomplished by comparing the retention times and the standard curves with the standards, respectively.

### 2.7 Determination of antioxidant capacity

The DPPH radical scavenging activity, ABTS^+^ radical scavenging activity, and hydroxyl radical scavenging activity of fig wines were determined by referring to previous reported methods (Sun, Chen, Feng, et al., 2024). The reactions were performed following the corresponding instructions of assay kits (Solarbio, Beijing, China), and the absorption of 517 nm, 734 nm, and 510 nm were determined by a microplate reader (SPARK 10 M, TECAN, Switzerland).

### 2.8 Determination of volatile compounds

The volatile compounds were extracted by headspace solid phase micro-extraction and detected by an Agilent 7890 gas chromatography–mass spectrometry (Agilent Technologies, CA, USA) equipped with an Agilent DB-5MS capillary column (30 m × 0.25 mm × 0.25 μm). Specifically, 5 mL of the sample was mixed with 10 μL of 2-methyl-3-heptanone (40.8 mg/L) and 2 g of sodium chloride. Then, the mixture was equilibrated at 40 °C for 20 min with agitation, and the fiber was inserted into the headspace for 20 min and desorbed into the GC injection port for 10 min. The technological parameters of gas chromatography–mass spectrometry were set as our previously described (Han et al., 2022). The volatile compounds were identified by comparing their mass spectra to the NIST 8.0 and Willey 7 standard libraries, and semi-quantified by calculating the relative peak area in relation to that of the internal standard. The odor activity value (OAV) was calculated as the ratio of the content of each volatile compound to its odor threshold.

### 2.9 *E*-nose and E-tongue analysis

Based on the method of Chen et al. (2023), the E-nose analysis was carried out using a PEN3 E-nose system (Airsense, Germany) coupled with 10 different metal sensors (W1C, W3C, W5C, W1S, W2S, W3S, W5S, W6S, W1W, W2W). In brief, 10 mL of the sample was added into a sealed headspace vial and incubated at 45 °C for 20 min. The E-nose detection program was set as follows: clean air was pumped into the gas path for 30 s, followed by a sample preparation time for 5 s and a collection time for 90 s with a flow rate of 200 mL/min. The E-nose array response values were obtained from the resistivity (measured in Ohms) of sensors.

According to the method of Chen et al. (2025), the E-tongue analysis was carried out using a SA402B taste-sensing system (Insent Co., Ltd., Tokyo, Japan) outfitted with a set of multichannel lipid/polymer membrane electrodes, which were assigned to characterize the sourness, sweetness, bitterness, saltiness, umami, and astringency of the fig wines. The E-tongue detection procedure was set as follows: stirring rate of 60 rpm; collection time of 120 s; and cleaning time of 30 s. Five repeated tests were carried out for each sample, and the middle three measurements were selected for raw data analysis.

### 2.10 Sensory analysis

Referring to our pervious method (Han et al., 2025), the sensory analysis was performed by a panel consisted of 10 well-trained members. The fig wine samples were rated on seven aroma aspects (floral, tropical fruit, stone fruits, berries, vanilla, herbal, and spice), ranging from 0 (very low intensity) to 5 (high intensity) points. All samples were presented to each panel member in duplicate and in a fixed order.

### 2.11 Statistical analysis

Data were expressed as the mean ± standard deviation in triplicate. The statistical analysis was conducted using Microsoft Excel 2010 (Microsoft, Redmond, WA, USA), employing a one-way analysis of variance (one-way ANOVA) followed by multiple comparative analysis using Duncan's test to assess significant differences (*P* < 0.05). Figures were drawn using Microsoft Excel 2010 (Microsoft, Redmond, WA, USA) and GraphPad Prism 8.0 software (GraphPad Software Inc., USA). Principal component analysis (PCA) and hierarchical clustering analysis (HCA) were visualized by SIMCA 14.0 (Umetrics AB, Sweden).

## 3 Results and discussion

### 3.1 Characterization of β-glucosidase and esterase activity of yeast strains

**Fig. 1:**
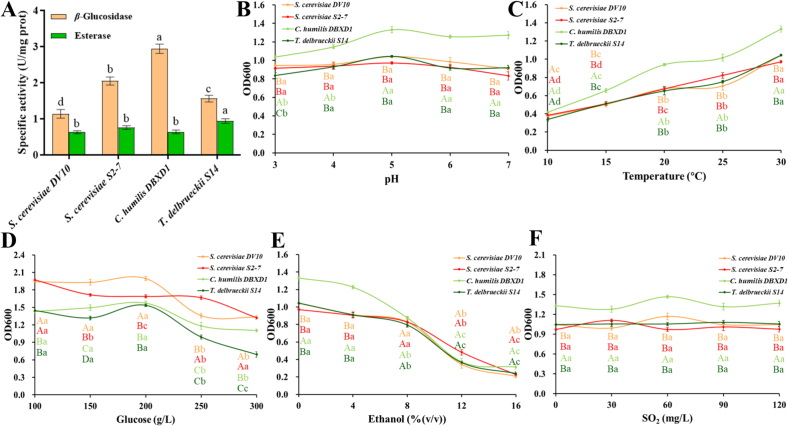
(A) *β*-glucosidase and esterase activity of yeast strains. Effects of (B) pH, (C) temperature, (D) glucose, (E) ethanol, and (F) SO_2_ on the growth activity of yeast strains. Different letters represent significant difference (*P* < 0.05).

The enzyme-producing characteristics of S2–7, DBXD1, and S14 were showed in Fig. 1A. All strains showed higher *β*-glucosidase activity than DV10, with the highest level of 2.94 ± 0.13 U/mg prot recording for DBXD1, which was higher than the *β*-glucosidase activity of *C. glabrata* D18 reported by Han et al. (2021). Moreover, the esterase activity was lower than that of *β*-glucosidase, which was agreement with the enzyme performance of yeast strains isolated from kiwifruit wine (Sun, Chen, Bi, et al., 2024). The highest esterase activity was found in S14 of 0.94 ± 0.06 U/mg prot, and the other two strains exhibited similar esterase activity to DV10. Overall, these three strains performed well in both enzyme assays. Then, the growth activity of these strains under oenological conditions was analyzed to evaluate their applicability in practical fermentation of fig wine. As shown in Fig. 1B-F, DBXD1 exhibited the greatest growth activity under the normal pH, temperature, and SO_2_ content of winemaking. All strains could thrive under conditions of higher SO_2_ (120 mg/L), lower pH value (3–4), and lower temperature (15–20 °C) as well as DV10, but their tolerance to higher glucose (250 and 300 g/L) was weaker than DV10 except for S2–7. Thus, considering the enzyme activity and environmental adaptability, these strains could be further utilized in the micro-vinification of fig wine.

### 3.2 The performance of high-enzyme producing yeast strains in fig wine fermentation

#### 3.2.1 Physiochemical properties of fig wines with different fermentation schemes

**Fig. 2:**
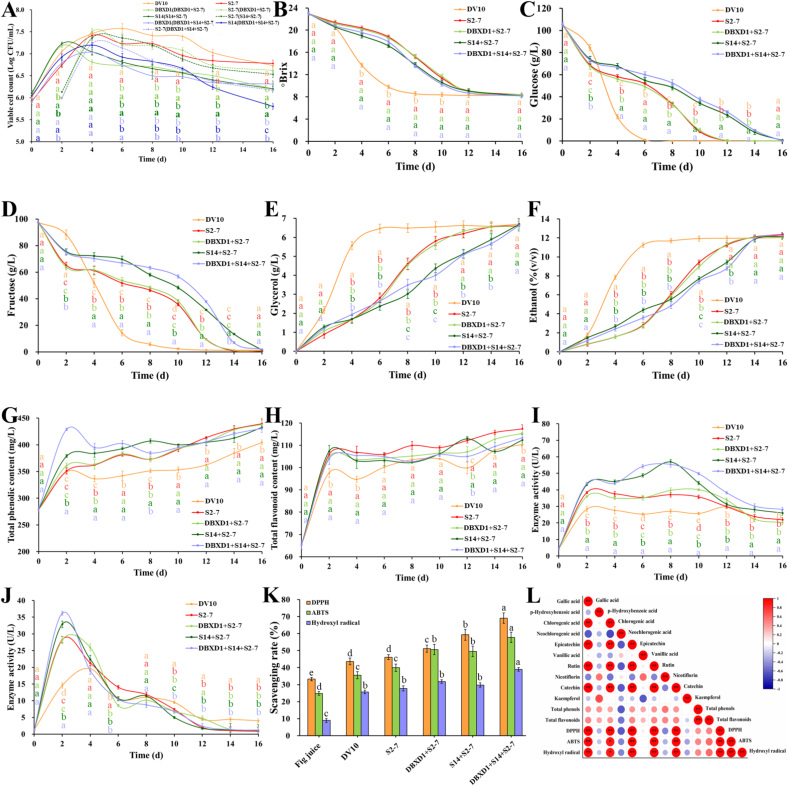
Changes of (A) viable cell count, (B) °Brix, (C) glucose, (D) fructose, (E) glycerol, (F) ethanol, (G) total phenolic content, (H) total flavonoid content, (I) *β*-glucosidase activity, (J) esterase activity, (K) antioxidant activity, and (L) the correlation between antioxidant activity and individual polyphenol content during the fermentation of fig wines with different inoculation schemes. Different letters represent significant difference (*P* < 0.05).

The growth dynamics of yeast is highly correlated with the flavor characteristics of fruit wines (Che et al., 2024). As shown in Fig. 2A, in the pure fermentations, DV10 and S2–7 performed similar growth dynamics, with rapid growth observed in the first 4 days, and a stable vitality maintained for 10 days. However, DV10 presented a higher viable cell count than that of S2–7, which was consistent with the faster fermentation rate of the DV10 group. In the sequential fermentations, S2–7 reached its maximum biomass on the 2nd day of inoculation, followed by gradual decrease until the end of fermentation. In the DBXD1 + S2–7 and S14 + S2–7 groups, DBXD1 and S14 reached their peak at 2 d, while these two strains reached their peak at 4 d in the DBXD1 + S14 + S2–7 group, which may be attributed to the competition between these two strains for sugar, oxygen uptake, and nutrients. It has been reported that non-*Saccharomyces* yeasts often play important roles in the early stage of sequential fermentation, and the populations of *S. cerevisiae* were always higher than that of non-*Saccharomyces* yeasts in the later fermentation stage (Benito et al., 2015; Li et al., 2022). This phenomenon was agreement with our results and may be due to the high tolerance of *S. cerevisiae* to ethanol (Li et al., 2022).

Monitoring basic physiochemical indicators (°Brix value, glucose, fructose, glycerol, and ethanol) was crucial for evaluating the quality of fig wines produced by different inoculation schemes. As depicted in Fig. 2B-F, the S2–7 group and the DBXD1 + S2–7 group exhibited similar fermentation kinetics, while the S14 + S2–7 group showed fermentation kinetics similar to the DBXD1 + S14 + S2–7 group. Reducing sugar is the main nutrient and energy source for yeast growth, which can be transferred into glycerol and ethanol via glycolysis and other metabolic pathways (Che et al., 2024). These results indicated that *S. cerevisiae* S2–7 might be the major sugar consumer in fermentation, and the inoculation of S14 may interfere with the sugar consumption efficiency of S2–7. The exceptionally high sugar content (21–24°Brix) of figs likely induced osmotic stress during the initial fermentation phase, delaying yeast adaptation and prolonging total fermentation time, a phenomenon documented in high-sugar grape musts and grape wines (Han et al., 2025). However, there was no significant difference in the residual sugar (0.47–1.96 g/L), glycerol (6.57–6.70 g/L), and ethanol (12.03–12.36%(*v*/v)) content among finial fig wines (Table 1). Besides, among the sequential fermentations, the S14 + S2–7 group and DBXD1 + S14 + S2–7 group showed slower fermentation rates than the DBXD1 + S2–7 group, which can be inferred that the diverse species of non-*Saccharomyces* yeasts possessed varying degrees of fermentation activity (Suzzi et al., 2012), which may have an impact on the nutritional and aroma characteristics of fig wines.

**Table 1:** Physicochemical parameters of fig wines fermented by different yeast strains.

		Fig wines				
Parameters	Fig juice	DV10	S2–7	DBXD1 + S2–7	S14 + S2–7	DBXD1 + S14 + S2–7
**Basic parameters**						
°Brix	23.0 ± 0.31^a^	8.4 ± 0.37^b^	8.4 ± 0.33^b^	8.4 ± 0.34^b^	8.2 ± 0.31^b^	8.2 ± 0.35^b^
pH	4.29 ± 0.12^a^	4.25 ± 0.16^a^	4.27 ± 0.14^a^	4.30 ± 0.13^a^	4.08 ± 0.11^b^	4.01 ± 0.14^b^
Titratable acidity (g/L)	7.04 ± 0.13^a^	5.15 ± 0.13^b^	4.70 ± 0.12^c^	4.70 ± 0.11^c^	4.32 ± 0.14^d^	4.17 ± 0.14^d^
Glucose (g/L)	105.37 ± 2.08^a^	N.D.	N.D.	N.D.	0.6 ± 0.03^b^	N.D.
Fructose (g/L)	97.47 ± 2.10^a^	1.28 ± 0.16^b^	0.47 ± 0.04^b^	1.16 ± 0.03^b^	1.36 ± 0.05^b^	1.93 ± 0.06^b^
Residual sugar (g/L)	202.84 ± 2.08^a^	1.28 ± 0.16^b^	0.47 ± 0.04^b^	1.16 ± 0.03^b^	1.96 ± 0.06^b^	1.93 ± 0.06^b^
Glycerol (g/L)	N.D.	6.70 ± 0.21^a^	6.60 ± 0.25^a^	6.57 ± 0.23^a^	6.69 ± 0.29^a^	6.63 ± 0.19^a^
Ethanol (%(v/v))	N.D.	12.03 ± 0.20^a^	12.36 ± 0.22^a^	12.09 ± 0.25^a^	12.18 ± 0.17^a^	12.24 ± 0.21^a^
Total phenols (mg/L)	279.28 ± 5.16^c^	404.50 ± 4.60^b^	439.75 ± 9.30^a^	438.15 ± 6.86^a^	432.90 ± 3.54^a^	430.60 ± 7.71^a^
Total flavonoids (mg/L)	65.25 ± 1.92^b^	105.40 ± 0.85^b^	117.40 ± 1.83^a^	115.40 ± 2.05^a^	112.40 ± 2.45^a^	113.40 ± 1.27^a^
L*	58.58 ± 2.17^b^	79.27 ± 2.15^a^	79.04 ± 2.26^a^	79.91 ± 2.03^a^	79.66 ± 2.17^a^	79.45 ± 2.11^a^
a*	18.95 ± 0.22^a^	7.89 ± 0.17^b^	7.33 ± 0.14^b^	7.17 ± 0.11^b^	6.00 ± 0.18^c^	6.38 ± 0.12^c^
b*	44.17 ± 1.73^b^	44.10 ± 1.82^b^	45.42 ± 1.95^a^	44.45 ± 1.70^b^	42.48 ± 1.99^c^	42.66 ± 2.02^c^
ΔE*	45.11 ± 1.17^a^	26.02 ± 0.92^c^	26.97 ± 0.83^b^	25.68 ± 0.79^c^	23.96 ± 0.80^d^	24.34 ± 0.10^d^
Simulated color						
**Polyphenols**						
Gallic acid (mg/L)	11.38 ± 1.13^b^	13.29 ± 1.29^b^	14.32 ± 1.10^b^	18.47 ± 1.58^a^	20.25 ± 1.74^a^	23.64 ± 1.62^a^
p-Hydroxybenzoic acid (mg/L)	7.62 ± 0.37^a^	6.09 ± 0.24^a^	7.83 ± 0.18^a^	7.50 ± 0.22^a^	7.19 ± 0.31^a^	7.06 ± 0.27^a^
Chlorogenic acid (mg/L)	12.99 ± 1.19^f^	20.77 ± 1.05^e^	39.91 ± 1.72^d^	59.42 ± 2.48^c^	66.17 ± 3.41^b^	89.77 ± 3.89^a^
Neochlorogenic acid (mg/L)	14.28 ± 1.01^a^	14.61 ± 1.17^a^	11.45 ± 1.25^a^	12.09 ± 1.37^a^	10.41 ± 0.97^a^	11.95 ± 1.12^a^
Epicatechin (mg/L)	18.94 ± 1.27^d^	24.60 ± 1.48^c^	25.11 ± 1.55^c^	34.11 ± 2.17^b^	36.63 ± 2.36^b^	46.27 ± 3.12^a^
Vanillic acid (mg/L)	7.18 ± 0.34^a^	9.02 ± 0.42^a^	8.67 ± 0.57^a^	8.96 ± 0.44^a^	7.90 ± 0.39^a^	7.53 ± 0.40^a^
Rutin (mg/L)	1.98 ± 0.12^d^	2.08 ± 0.21^d^	3.10 ± 0.17^c^	4.07 ± 0.25^b^	4.53 ± 0.21^b^	5.95 ± 0.30^a^
Nicotiflorin (mg/L)	15.76 ± 1.27^a^	16.10 ± 1.48^a^	17.56 ± 1.52^a^	17.37 ± 1.29^a^	15.44 ± 1.43^a^	15.42 ± 1.52^a^
Catechin (mg/L)	17.73 ± 1.47^f^	31.91 ± 2.29^e^	40.73 ± 2.58^d^	61.39 ± 3.16^c^	68.69 ± 3.27^b^	79.14 ± 3.83^a^
Kaempferol (mg/L)	1.83 ± 0.17^a^	0.91 ± 0.07^a^	1.11 ± 0.12^a^	1.11 ± 0.13^a^	1.14 ± 0.15^a^	1.02 ± 0.09^a^
Total polyphenols (mg/L)	109.69 ± 4.38^e^	139.37 ± 5.27^d^	169.80 ± 7.94^c^	224.48 ± 11.38^b^	238.35 ± 9.27^b^	287.74 ± 14.75^a^
**Organic acids**						
Citric acid (g/L)	3.96 ± 0.21^a^	2.50 ± 0.15^b^	2.52 ± 0.17^b^	2.39 ± 0.22^b^	2.48 ± 0.19^b^	2.48 ± 0.24^b^
Tartaric acid (g/L)	0.98 ± 0.10^c^	1.17 ± 0.27^c^	2.65 ± 0.06^b^	3.53 ± 0.12^a^	3.02 ± 0.09^a^	3.91 ± 0.11^a^
Malic acid (g/L)	4.08 ± 0.31^a^	2.67 ± 0.21^b^	2.86 ± 0.22^b^	2.76 ± 0.25^b^	2.03 ± 0.24^c^	1.83 ± 0.22^c^
Succinic acid (g/L)	N.D.	9.99 ± 0.12^a^	6.42 ± 0.43^b^	3.16 ± 0.27^c^	3.85 ± 0.58^c^	5.98 ± 0.48^b^
Lactic acid (g/L)	42.61 ± 1.52^a^	7.96 ± 1.03^c^	9.39 ± 1.17^c^	17.86 ± 0.62^b^	16.40 ± 0.46^b^	16.59 ± 0.42^b^
Acetic acid (g/L)	0.53 ± 0.15^b^	1.13 ± 0.17^a^	1.18 ± 0.11^a^	1.06 ± 0.10^a^	1.12 ± 0.09^a^	1.18 ± 0.13^a^
Total organic acids (g/L)	52.15 ± 3.27^a^	24.43 ± 2.18^c^	25.02 ± 2.86^c^	30.76 ± 1.59^b^	28.90 ± 1.74^b^	30.96 ± 1.32^b^
**Amino acids**						
Aspartic acid (mg/L)	153.13 ± 3.27^a^	101.58 ± 2.48^b^	103.92 ± 2.73^b^	72.46 ± 1.30^c^	72.13 ± 1.58^c^	62.53 ± 1.42^c^
Threonine (mg/L)	1895.42 ± 5.15^a^	787.04 ± 4.72^c^	845.15 ± 4.19^b^	688.99 ± 4.15^d^	695.97 ± 4.36^d^	495.34 ± 3.18^e^
Serine (mg/L)	N.D.	N.D.	N.D.	N.D.	N.D.	N.D.
Glutamic acid (mg/L)	N.D.	104.70 ± 4.29^a^	99.60 ± 4.93^a^	61.45 ± 3.15^b^	65.22 ± 3.42^b^	61.05 ± 2.17^b^
Glycine (mg/L)	34.63 ± 2.05^a^	36.33 ± 1.41^a^	39.95 ± 1.25^a^	37.98 ± 1.83^a^	38.82 ± 1.72^a^	40.02 ± 2.38^a^
Alanine (mg/L)	398.47 ± 4.75^a^	231.51 ± 2.78^b^	235.60 ± 3.49^b^	211.11 ± 3.15^b^	210.17 ± 3.74^b^	154.32 ± 4.29^c^
Cystina (mg/L)	N.D.	N.D.	N.D.	12.55 ± 0.75^a^	12.23 ± 0.73^a^	7.25 ± 0.42^b^
Valine (mg/L)	116.36 ± 5.41^a^	79.88 ± 3.62^b^	82.25 ± 4.38^b^	39.62 ± 1.73^c^	41.01 ± 1.45^c^	17.11 ± 1.29^d^
Methionine (mg/L)	25.49 ± 1.47^a^	10.43 ± 1.31^b^	9.85 ± 1.25^b^	0.93 ± 0.05^c^	1.70 ± 0.09^c^	2.91 ± 0.12^c^
Isoleucine (mg/L)	70.01 ± 3.27^a^	53.72 ± 2.58^b^	51.52 ± 3.17^b^	10.71 ± 1.24^c^	13.04 ± 0.79^c^	4.24 ± 0.32^d^
Leucine (mg/L)	181.62 ± 4.28^a^	98.91 ± 3.84^b^	84.21 ± 3.17^c^	6.56 ± 0.71^d^	7.10 ± 0.82^d^	11.89 ± 1.25^d^
Tyrosine (mg/L)	145.46 ± 3.75^a^	73.32 ± 2.46^b^	76.34 ± 2.77^b^	46.94 ± 2.19^d^	47.50 ± 2.33^d^	55.17 ± 2.69^c^
Phenylalanine (mg/L)	202.72 ± 4.86^a^	79.08 ± 3.17^b^	78.21 ± 3.49^b^	23.90 ± 1.62^d^	24.65 ± 1.47^d^	35.19 ± 2.15^c^
Lysine (mg/L)	145.08 ± 3.46^a^	31.99 ± 1.45^b^	36.56 ± 1.72^b^	1.65 ± 0.16^d^	6.04 ± 0.83^cd^	12.74 ± 1.27^c^
Histidine (mg/L)	47.24 ± 2.46^a^	18.94 ± 1.43^b^	16.96 ± 1.29^b^	2.81 ± 0.12^c^	1.05 ± 0.17^c^	5.03 ± 0.38^c^
Arginine (mg/L)	136.63 ± 4.83^a^	N.D.	N.D.	3.70 ± 0.17^b^	N.D.	6.43 ± 0.39^b^
Proline (mg/L)	814.28 ± 5.92^a^	409.07 ± 4.27^b^	416.67 ± 4.90^b^	433.64 ± 4.18^b^	432.40 ± 3.66^b^	419.20 ± 3.15^b^
Total amino acids (mg/L)	4366.53 ± 6.25^a^	2116.51 ± 6.70^b^	2176.79 ± 6.55^b^	1655.02 ± 5.83^c^	1669.05 ± 4.91^c^	1390.41 ± 5.48^d^

N.D.: not detected or below limit of quantitation. Different superscripts in the same column indicated significant difference in the concentration of the measured index, *P* < 0.05.

The pH values, titratable acidity, and color attributes of fig wines were shown in Table 1. The pH values of the S14 + S2–7 and DBXD1 + S14 + S2–7 groups were significantly lower than those of the other three groups. Similarly, these two groups exhibited the lowest titratable acidity, which might be correlated with higher malic acid consumption by *T. delbrueckii* S14. Several studies have reported that the inoculation of *T. delbrueckii* led to significant decrease in titratable acidity and malic acid content compared to the pure *S. cerevisiae* inoculation (Belda et al., 2015; Wang et al., 2024). Color is considered the most direct way to determining consumers' acceptance of fruit wines. The L* value of all groups were higher than that of fig juice, indicating that both pure and sequential inoculation had positive effects on the brightness of fig wines. The a*, b*, and ΔE* values were significantly lower in groups involving S14 than those in the other three groups, and the differences were sufficient to be visible with the naked eye (net difference between Δ E * > 2) (Hensel et al., 2023).

Polyphenols are regarded some of the most important quality indicators of fruit wines, which can affect sensory properties and provide various health benefits (Peng et al., 2025). As shown in Fig. 2G-H, the content of total phenols and total flavonoids increased dramatically in the first two days of fermentation, then slightly increased until the fermentation was completed. The groups involving high enzyme-producing strains showed higher total phenol and total flavonoid content than the DV10 group, indicating that these strains may have the ability to produce polyphenols during fig wine fermentation. Several studies have suggested that the change in polyphenol content could be correlated with the activity of *β*-glucosidase (Lacchini et al., 2023). As shown in Fig. 2I, the activity of *β*-glucosidase increased rapidly in the first two days of fermentation and continued to maintain a stable vigor until the 12th d of the fermentation. Moreover, the activity of *β*-glucosidase in groups involving high enzyme-producing strains was significantly higher than that in the DV10 group, which was consistent with the differences in total phenol and flavonoid content. The esterase is another important index affecting the mouthfeel and flavor of fruit wines (Sun, Chen, Bi, et al., 2024). As shown in Fig. 2J, the activity of esterase reached it maximum after 2 days of fermentation except for the DV10 group, which showed the greatest activity after 4 days of fermentation. Besides, the maximum esterase activity in groups involving high enzyme-producing strains was higher than the DV10 group, suggesting the great potential of these strains in improving the sensory characteristics of fig wines. Notably, the enzyme activities reported in this study were measured in cell-free supernatants and therefore represent primarily extracellular enzyme pools. This distinction is important because only enzymes that are secreted into the fermentation medium or localized in the periplasmic space can directly access extracellular substrates such as glycosidic aroma precursors or esters (Kong et al., 2019). Nevertheless, future studies should additionally measure intracellular enzyme activity in cell extracts to provide more direct evidence of the strains' potential to modify aromatic compounds during fermentation.

#### 3.2.2 Polyphenols and antioxidant activity of fig wines with different fermentation schemes

The concentration variation of polyphenols in fig wines with different fermentation schemes were shown in Table 1. Compared with the fig juice, the total polyphenol content in fig wines were increased significantly, and the sequential fermentation obtained higher yields. This result was confirmed the previous finding that some non-*Saccharomyces* yeasts could enhance polyphenol content and consequently improve the sensory attributes of apple wines (Wei et al., 2019). Among individual polyphenols, chlorogenic acid, epicatechin, and catechin were the most abundant, and their contents were higher in sequential fermentations than in pure fermentations, indicating that high *β*-glucosidase activity in sequential inoculation treatments likely liberated phenolic aglycones from fig-derived glycosidic precursors, contributing to the volatile phenol pool detected in fig wines. In agreement with the study of Anjos et al. (2024), the difference in polyphenol content between these two fermentation patterns can be attributed to the differences in enzyme activity (pectinases, esterases, proteases, and *β*-glucosidase) of yeasts, which affect the release of phenolic compounds such as anthocyanins and flavonols.

The DPPH, ABTS^+^, and hydroxyl radical scavenging rates of fig wines were illustrated in Fig. 2K. It was observed that the sequential fermentation attained higher DPPH and ABTS^+^ radical scavenging rates than single fermentation, thus confirming its potential to improve the antioxidant activity of fig wines. However, the difference of hydroxyl radical scavenging rates between these two inoculation schemes was relatively subtle, with the highest hydroxyl radical scavenging rate in the DBXD1 + S14 + S2–7 group. Furthermore, correlation analysis was applied to investigate the relationship between different polyphenols and antioxidant activity. As shown in Fig. 2L, there was a positive correlation between antioxidant activity parameters and gallic acid, chlorogenic acid, epicatechin, rutin, and catechin, while other monomer polyphenols exhibited negative correlations with these parameters, which was consistent with the results reported by Sun et al. (2022) and Anjos et al. (2024). The strong correlation between rutin and catechin implied that the synthetic pathway of these two polyphenols may be upstream or downstream of a certain metabolic pathway (Jiang et al., 2024). Besides, gallic acid, chlorogenic acid, epicatechin, rutin, and catechin were also positively correlated.

#### 3.2.3 Organic acids of fig wines with different fermentation schemes

Organic acids play an important role in determining the color, flavor, and chemical stability of fruit wines (Cui et al., 2024). The results of organic acids in fig wines were shown in Table 1. The total organic acid content of all fig wines decreased, and the sequential fermentations showed higher total content than the pure fermentations. The contents of citric acid and malic acid in all groups also decreased significantly after fermentation, which may be attributed to yeast degradation of these two acids as carbon sources (Vilela, 2017). Lactic acid was the major acid in fig juice and fig wines, with the highest content in sequential fermentations, indicating that the sequential inoculation would be a great technique to improve the softness and balanced taste of fig wines (Sun, Chen, Bi, et al., 2024).Succinic acid is an acid with a bitter and salty taste at high levels (Restuccia et al., 2017), while high content of tartaric acid had a positive impact on the mouthfeel perception of fruit wines by reducing astringency and enhancing acidity (Cui et al., 2024). The groups fermented with high enzyme-producing strains showed higher tartaric acid content and lower succinic acid content compare to the DV10 group, indicating that the high enzyme-producing strains had certain ability to weaken the adverse effects caused by succinic acid and be in favor of the mouthfeel perception of fig wines. Besides, all fermentations produced acetic acid in an acceptable rang (1.06–1.18 g/L), avoiding bringing unpleasant odors to fig wines (< 1.2 g/L) (Gamboa et al., 2019).

#### 3.2.4 Amino acids of fig wines with different fermentation schemes

Amino acids play an important role in synthetizing higher alcohols, esters, and polyphenolic compounds, which can affect the sensory characteristics and nutritional value of fruit wines (Scutarașu et al., 2022). The results of the 17 types of free amino acids in fig wines were shown in Table 1. The contents of most amino acids in fig wines, except for glutamic acid, glycine, and cystina, were lower than those in fig juice, whether under pure fermentations or sequential fermentations. Among these, the contents of tyrosine and phenylalanine, acting as precursors of polyphenols, decreased dramatically after fermentation, indicating the great potential of high enzyme-producing strains to produce polyphenols (Ma et al., 2022). Besides, the contents of branched-chain amino acids (leucine, isoleucine, and valine) in sequential fermentations were lower than the pure fermentations, which suggested that the involvement of non-*Saccharomyces* yeasts could promote the transformation of branched-chain amino acids into higher alcohols and esters (Peng et al., 2025), thereby enhancing the distinctive flavor of fig wines. Furthermore, the sequential fermentations showed higher consumption of total amino acids compared to the pure fermentations, which can be inferred that the nitrogen metabolism pathways involved in alcoholic fermentation differed between these two fermentation schemes (Anjos et al., 2024). However, the amino acid consumption profile of each strain was hard to elucidate due to the sharing of intermediates.

### 3.3 Aroma profiles of fig wines with different fermentation schemes

#### 3.3.1 Categorization-based analysis of volatile compounds

The profiles of volatile compounds in fig wines were summarized in Table S1 and the key compounds with an OAV > 1.0 were exhibited in Table S2. A total of 68 volatile compounds belonging to five chemical families were identified and semi-quantified in fig wines. Among these, esters, high alcohols, and terpenoids were the group with the highest number of VOCs important for fig wine aroma. In details, a total of 35 esters were detected, including 9 acetate esters, 13 ethyl esters, and 13 other esters. Ethyl acetate (23,136.34–49,845.49 μg/L) was the most abundant acetate ester, while octanoic acid, ethyl ester (25,223.60–98,788.16 μg/L) and decanoic acid, ethyl ester (33,521.49–93,036.53 μg/L) were the ethyl esters with the highest content in sequential fermentation groups and single fermentation groups, respectively. Besides, the content of these three esters was higher in sequential inoculation than that in pure inoculation, indicating the great potential of sequential inoculation in enhancing fruity and floral aromas of fig wines (Sun, Chen, Feng, et al., 2024). Similarly, isoamyl acetate and hexanoic acid, ethyl ester, which was the acetate ester and ethyl ester with the highest OAVs, respectively, showed higher content in sequential fermentation groups, providing fruity aromas like banana, green apple, and pineapple to fig wines (Wang et al., 2025). Furthermore, except for the esters mentioned above, the contents of acetic acid, 2-phenylethyl ester, butanoic acid, ethyl ester, heptanoic acid, ethyl ester, ethyl 9-decenoate, octanoic acid, methyl ester, and octanoic acid, 3-methylbutyl ester in the sequential fermentations were higher than those in the pure fermentations, indicating the high ability of DBXD1 and S14 to produce these esters and endow fig wines with sensed rose, pineapple, cheese, and citrus aromas (Li et al., 2022). Notably, compared with single inoculation of non-*Saccharomyces* yeasts, DBXD1 and S14 co-inoculation showed higher levels of ethyl acetate, isoamyl acetate, acetic acid, hexyl ester, butanoic acid, ethyl ester, and butanoic acid, methyl ester, indicating the co-inoculation of DBXD1 and S14 had great potential in imparting fig wines with sensed apple, pineapple, banana, and pear aromas (Li et al., 2022).

High alcohols were another main group of volatile compounds in fig wines, with the total content of 153.41–214.31 mg/L, which could enhance the desirable aroma complexity of fig wines (< 300 mg/L) (Swiegers & Pretorius, 2005). Among these, 3-methyl-1-butanol exhibited the highest content in all groups, and its contribution to banana, mellow, whiskey characteristics could be perceived even at high odor threshold (OAVs: 2.83–4.95) (Wang et al., 2025). In addition, the content of 2-nonanol in sequential inoculation groups was higher than that in single inoculation groups, indicating the involving of DBXD1 or S14 may increase the sensed citrus and cheese aromas of fig wines (Li et al., 2022; Wang et al., 2025). Benzyl alcohol and phenylethyl alcohol were also important high alcohols in fig wines. The pure fermentations possessed higher benzyl alcohol content than the sequential fermentations, whereas the opposite result was observed in phenylethyl alcohol content. These results indicated that single inoculation of DV10 or S2–7 may bring inevitable bitterness of almonds, while sequential inoculation of DBXD1 or S14 may reduce this bitterness and enhance rose aromas of fig wines (Wang, Qi, et al., 2023). Besides, 3-methyl-2-hexanol was present in a content exceeding its odor threshold in the S2–7 group, indicating the great ability of S2–7 to produce this compound and impart fruity aromas to fig wines (Jiang et al., 2024).

In terms of acids, acetic acid and octanoic acid were the major acids in fig wines, but their OAVs were relatively low (0.04–3.72) because of high odor thresholds (Jiang et al., 2024; Wang, Qi, et al., 2023). Besides, octanoic acid was present in a content exceeding its odor threshold in sequential inoculation groups, which may bring fatty, waxy, and cheese odors to fig wines (Jiang et al., 2024). Regarding aldehydes and ketones, the most important were nonanal and 2-nonanone, which could be perceived as rose, citrus, and herbal aromas in all fermentations (Wang et al., 2024), with the highest content in the DBXD1 + S14 + S2–7 group. Besides, terpenoids were also an important group that imparted fig wines with rich floral and fruity aromas like rose, citrus, and lemony due to their low odor thresholds (Wang et al., 2025; Wang, Qi, et al., 2023). In this study, linalool exhibited the highest content in all groups, providing rose, citrus, lavender, and lemony aromas to fig wines (Wang et al., 2025). The contents of citronellol, rose oxide and β-damascenone in single fermentations were higher than that in sequential fermentations, while the opposite results were obtained for α-terpineol, indicating the great potential of DV10 and S2–7 in enriching rose, green, baked apple-like, and grape juice-like aromas of fig wines. While less studied than grapes, figs contain various terpene glycosides and carotenoid-derived compounds that serve as potential aroma precursors. The yeast strains with high *β*-glucosidase activity would hydrolyze these glycosides, releasing free terpenes (e.g., linalool, citronellol, and α-terpineol) and norisoprenoids (e.g., β-ionone and β-damascenone), respectively. Notably, figs contain high levels of pectin (approximately 0.7% fresh weight basis, depending on cultivar and ripeness), which can lead to colloidal instability, haze formation, and filtration difficulties in the finished wine (Gao et al., 2024). The decomposition of pectin can liberate entrapped glycosidic bound terpenes and norisoprenoids, making them available for catalytic hydrolysis by *β*-glucosidase to free aroma-active forms. Future optimization could explore pectinase enzyme treatments prior to fermentation to improve clarity and sensory perception of fig wines.

#### 3.3.2 Statistical analysis of volatile compounds

**Fig. 3:**
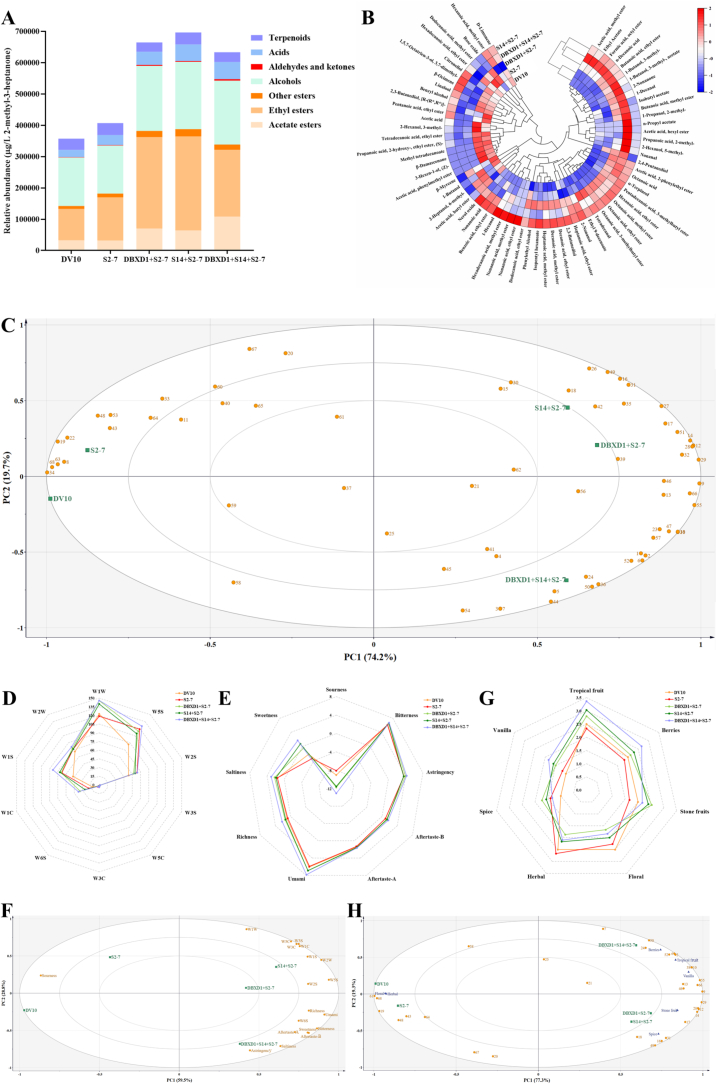
(A) The relative abundant of different types of volatile compounds in fig wines. (B) Clustering heatmap and (C) PCA plot of volatile compounds in fig wines. Radar charts of (D) E-nose data and (E) E-tongue data of fig wines. (F) PCA plot of E-nose and E-tongue profiles of fig wines. (G) Aroma radar map of sensory evaluation of fig wines. (H) The PLSR analysis based on the aroma intensities and the contents of volatile compounds. Different letters represent significant difference (*P* < 0.05). Compound numbers in figure C and H are available in Table S1.

To clarify the impacts of these three high enzyme-producing yeasts on the aroma profiles of fig wines, a variety of statistical analysis were carried out. As shown in Fig. 3A, ethyl esters and higher alcohols were the classes with the highest total content, followed by acetate esters and terpenoids. The sequential fermentations possessed higher content of total esters, alcohols, aldehydes, ketones, and acids than the pure fermentations, indicating that the inoculation of high enzyme-producing non-*Saccharomyces* strains would be a potential strategy to obtain distinctive fig wines with rich fruity and floral flavors. Furthermore, the S2–7 group showed higher total ester and terpenoid content than the DV10 group, which could be attributed to the higher ability of S2–7 to produce *β*-glucosidase and esterase during fermentation. Besides, the content of total acid and terpenoid in the S14 + S2–7 group was significantly higher than that in the DBXD1 + S2–7 group, while the opposite results were observed in the total aldehydes and ketone content, suggesting that S14 may be a more suitable and efficient high enzyme-producing strain in enhancing floral aromas of fig wines in oenological practice.

Then, the volatile compounds were carried out HCA, and the clustering heatmap was exhibited in Fig. 3B. The fig wines can be classified into two major groups based on the strength of their correlations. The first cluster grouped the two single fermentations, and the second cluster grouped fig wines produced by sequential inoculation. The S14 + S2–7 group did not group with the other two sequential fermentations until the distance scale reached 10.51. Moreover, the sequential fermentations were separated from single fermentations, and the aroma of sequential fermentations was enhanced to some extent, indicating that sequential inoculation of these two high enzyme-producing non-*Saccharomyces* yeasts played an important role in improving the aroma type and content of fig wines.

Afterwards, PCA was conducted to clarify the flavor features of fig wines with different fermentation schemes. As depicted in Fig. 3C, the cumulative total variability of the first two principal components was 93.9% (74.2% and 19.7% for PC1 and PC2, respectively). The DV10 and S2–7 groups were associated to negative PC1, that discriminated these two groups from the sequential fermentation groups. Besides, the S2–7 and DV10 groups located closely in the negative PC1 direction, which scored high relative correlations with tetradecanoic acid, ethyl ester, benzyl alcohol, acetic acid, and linalool. Similarly, the S14 + S2–7 group and the DBXD1 + S2–7 group dispersed at the same quadrant and located closely in the positive PC1 direction, which were characterized by higher levels of hexanoic acid, ethyl ester, octanoic acid, ethyl ester, decanoic acid, ethyl ester, ethyl 9-decenoate, 3-methyl-1-butanol, phenylethyl alcohol, and octanoic acid. It was worth noting that the DBXD1 + S14 + S2–7 group was clearly separated from all other groups along the PC2 direction, which mainly due to high values of components of the negative PC2, like ethyl acetate, n-propyl acetate, isoamyl acetate, 2-methyl-1-propanol, and 3-methyl-1-butanol. Esters conveyed intense fruity aroma like apple, banana, pear, and pineapple (Wang et al., 2025), and 2-methyl-1-propanol, 3-methyl-1-butanol, and phenylethyl alcohol also presented fruity and floral aromas like banana, rose, and citrus (Wang, Qi, et al., 2023). Thus, it could be inferred that compared with the single inoculation, the sequential inoculation of high enzyme-producing strains could enhance the typicality and complexity of the fruity and floral aromas in fig wines. From the perspective of industrial application, despite sequential inoculation adds operational costs compared to conventional single-strain fermentation, the enhanced aroma complexity and sensory profile we demonstrated could position fig wine as a premium product, potentially justifying higher market prices that offset increased production costs. Besides, factors such as oxygen transfer rates, mixing dynamics, and temperature control differ significantly with scale and can profoundly impact yeast performance and the success of sequential inoculation strategies. Thus, pilot-scale validation was recommended as an essential prerequisite before full industrial adoption.

#### 3.3.3 Sensory property analysis

The *E*-nose and E-tongue are crucial for rapidly and objectively analyzing the aroma and taste characteristics of fig wines with different inoculation schemes, respectively. As shown in Fig. 3D, the sequential fermentations exhibited higher signal intensities in W1W and W1C sensors compared to the pure fermentations, indicating that the sequential inoculation was sensitive to inorganic sulfides and aromatics. Besides, the DBXD1 + S14 + S2–7 group showed higher response values in the W5S, W1C, W1S, and W2S sensors than the other two sequential inoculation groups, indicating that higher levels of nitrogen oxides, short-chain alkanes, aromatics, and alcohols presented when D21 and X6 were co-inoculated. Meanwhile, the signal intensities of the other sensors (W3C, W5C, W3S, and W6S) were low and showed slight variation, likely due to the low content of long-chain alkanes, hydrides, and alkane aromatic compounds in all groups.

The taste profiles of fig wines were showed and differentiated based on the E-tongue analysis (Fig. 3E). The probes showed significant responses to the sourness, bitterness, astringency, and umami of fig wines. The DBXD1 + S14 + S2–7 group showed the highest response values in umami, richness, saltiness, and sweetness sensors, while displaying lowest response values in sourness sensor. Besides, the sequential fermentations yielded higher richness, umami, and sweetness values and lower sourness values compared to the single fermentations. These results indicated that the sequential inoculation could imparted richness, umami, and sweetness to fig wines, and co-inoculation of these two high enzyme-producing non-*Saccharomyces* strains further improved the taste to some extent.

The PCA model was further applied to distinguish different fig wines based on the E-nose and E-tongue analysis. As displayed in Fig. 3F, the total explained variance is 88.3% (PC1 at 59.5% and PC2 at 28.8%). The DV10 and S2–7 groups located closely in the negative PC1 direction, exhibiting strong correlations with sourness and W1W sensors. The DBXD1 + S2–7 group exhibited positive correlations with aftertaste-A, umami, and richness sensors, while the S14 + S2–7 group showed positive correlations with W5C, W3C, W1C, W1S, W2W. The result suggested that despite being located in the same quadrant, these two groups were different in their flavor characteristics. Moreover, the DBXD1 + S14 + S2–7 group was located in the fourth quadrant solely, potentially contributing to the W6S, astringency, aftertaste-B, saltiness, and sweetness sensors. Although the E-nose and E-tongue can effectively identify the overall flavor profiles of fig wines, they cannot provide more detailed information about differential VOCs. Thus, a more in-depth investigation was conducted on the correlations between the differential VOCs (OAV > 1.0) and sensory attributes of fig wines using sensory evaluation and PLSR (partial least square regression) analysis.

As shown in Fig. 3G and H, the inoculation patterns and yeast strains had significant effects on the sensory properties of fig wines. The total explained variance is 96.6% (PC1 at 77.3% and PC2 at 19.3%). The DV10 and S2–7 groups located closely in the negative PC1 direction, exhibiting strong correlations with floral and herbal aromas. The DBXD1 + S2–7 group and the S14 + S2–7 group were located in the same quadrant, exhibiting positive correlations with stone fruit and spice aromas. Moreover, the DBXD1 + S14 + S2–7 group was located in the first quadrant solely, potentially contributing to the berries, tropical fruit, and vanilla aromas. In comparison of pure DV10 inoculation, fig wines from pure S2–7 inoculation exhibited more pronounced vanilla, spice, and herbal aromas, which may be attributed to the synthetic effects of tetradecanoic acid, ethyl ester, benzyl alcohol, and linalool. Sequential fermentations ranked significantly higher in terms of tropical fruit, berries, stone fruits, and vanilla aromas compared to pure fermentations, which may be attributable to the participation of these three high enzyme-producing strains. Notably, fig wines from the DBXD1 + S14 + S2–7 group showed the highest intensity of tropical fruit, berries, and vanilla aromas, which could be attributed to the pleasant floral and fruity aromas produced by ethyl acetate, isoamyl acetate, butanoic acid, ethyl ester, 3-methyl-1-butanol, nonanal, and 2-nonanone. Compared with the typical grape wines we previously reported, these fig wines exhibited more pronounced floral and tropical fruit aromas, but the vanilla and spice aromas were slightly inferior (Han et al., 2021; Han et al., 2025). Therefore, it has been shown that the indigenous high enzyme-producing non-*Saccharomyces* yeast strains (DBXD1 and S14) could effectively improve the flavor profiles of fig wines in terms of esters and alcohols, thereby increasing typical floral and fruity aromas. In the future, it is necessary to perform controlled comparative fermentations using these high enzyme-producing yeast strains and fermentation conditions across multiple fruit substrates, which would isolate substrate-specific effects on yeast metabolism and flavor development, enabling rigorous identification of fig-unique metabolic outcomes. Besides, the sensory comparison using trained panels and standardized descriptive analysis across multiple fruit wines would validate and extend our preliminary sensory findings.

### 3.4 Correlation analysis of key metabolites and enzyme activity

**Fig. 4:**
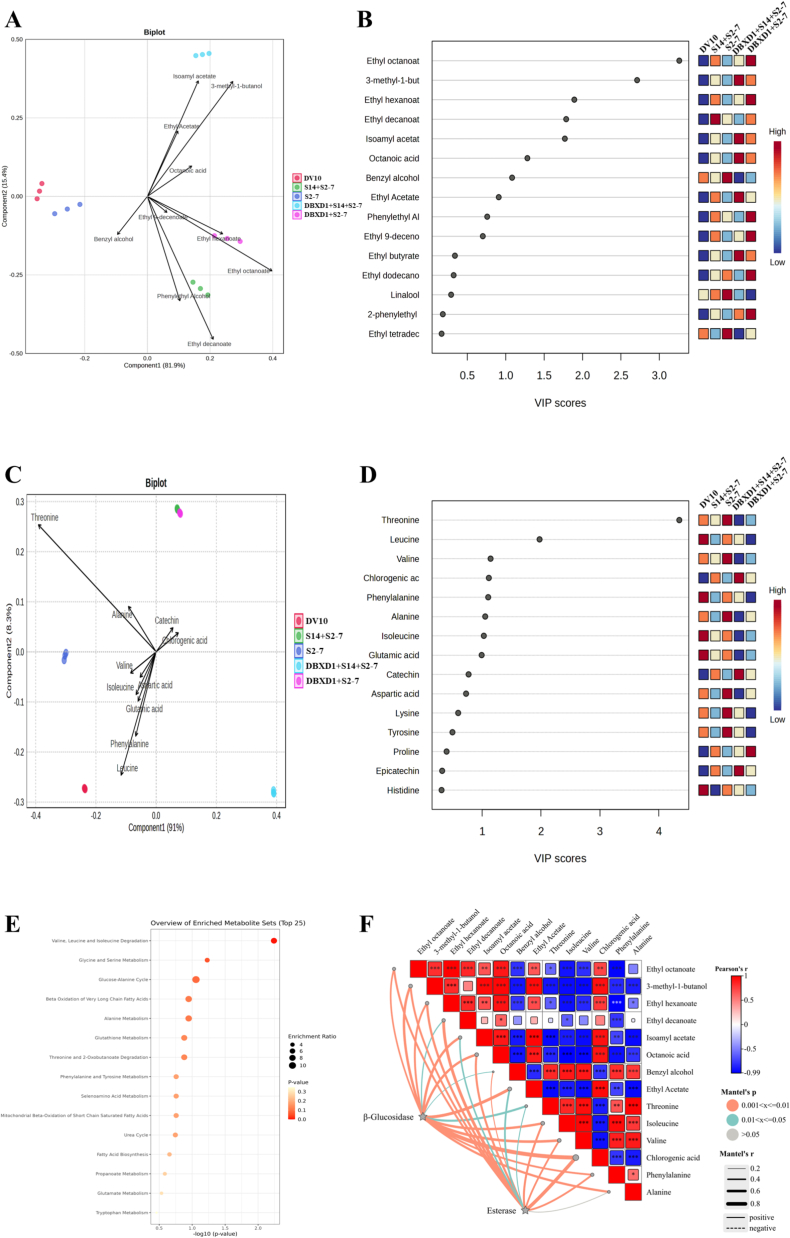

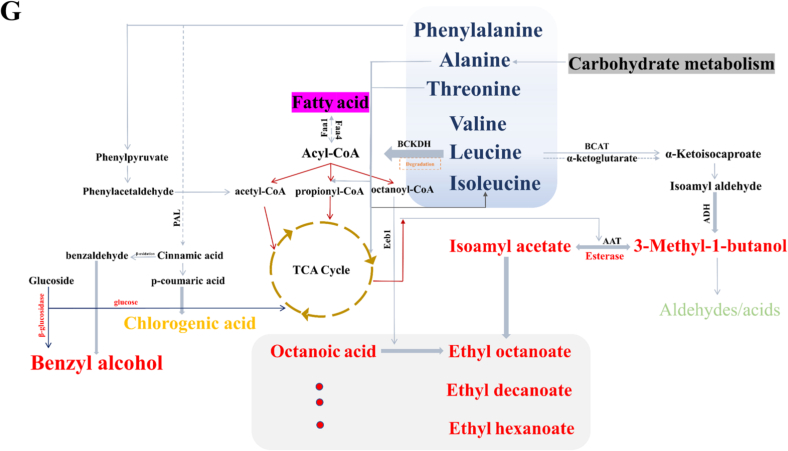
(A) PLS-DA biplot and (B) variable importance in projection (VIP) scores of differential volatile compounds in fig wines. (C) PLS-DA biplot and (D) variable importance in projection (VIP) scores of differential amino acids in fig wines. (E) Metabolite pathway analysis based on KEGG enrichment analysis. (F) Mantel test between enzyme activity and metabolites. (G) The potential metabolic pathways of differential characteristic flavor compounds.

To differentiate the key VOCs among fig wines with different fermentation schemes, PLS-DA analysis was performed on the VOCs with OAV > 1.0. As shown in Fig. 4A and B, the key VOCs were identified as benzyl alcohol, octanoic acid, isoamyl acetate, ethyl decanoate, ethyl hexanoate, 3-methyl-1-butanol, and ethyl octanoate (VIP score > 1.0). Similarly, we performed PLS-DA analysis on amino acids. As shown in Fig. 4C and D, the key amino acids were identified as threonine, leucine, valine, chlorogenic acid, phenylalanine, alanine, and isoleucine (VIP score > 1.0). We proposed that the higher availability of branched-chain amino acid precursors in fig juice directly enhanced the production of their corresponding higher alcohols and esters via the Ehrlich pathway. This explained the exceptionally high OAV values (>1.0) observed for these compounds in our fig wines-a fig-specific metabolic interaction that would not occur to the same extent in grape must. Furthermore, pathway analysis revealed major enrichment of differential metabolites in fig wines (Fig. 4E). Fifteen pathways that play important roles in the overall metabolic network were identified and were mainly related to valine, leucine and isoleucine degradation, glycine and serine metabolism, and glucose-alanine cycle. Then, the Mantel test was performed to determine the relationship between enzyme activities and the key metabolites. Mantel test showed that the *β*-glucosidase and esterase activity exhibited significant correlations with VOCs (benzyl alcohol, octanoic acid, isoamyl acetate, ethyl decanoate, ethyl hexanoate, 3-methyl-1-butanol, ethyl octanoate, and ethyl acetate) and amino acids (threonine, valine, chlorogenic acid, phenylalanine, and isoleucine) (Fig. 4F). Furthermore, the metabolic network of the key flavor metabolites was reconstructed by PICURST2 based on the KEGG database (Fig. 4G). The TCA cycle was the pivotal pathway of biological metabolism, and its metabolites can act as precursors to participate in the anabolism of amino acids and fatty acids (Aboulfazli et al., 2016). Moreover, fatty acids underwent β-oxidation to generate acyl-CoA, which subsequently involved in the TCA cycle. The contents of 3-methyl-1-butanol and benzyl alcohol in fig wines were relatively high, which might be related to the degradation of isoleucine, valine, leucine, and phenylalanine during the fermentation process. This may be the primary reason that all amino acids experienced a significant decrease during the fermentation of fig wines. Octanoic acid was generated through fatty acid biosynthesis pathway, and isoamyl acetate and ethyl esters (ethyl decanoate, ethyl hexanoate, ethyl octanoate, and ethyl acetate) were produced mainly by glycerolipid metabolism pathway under the action of esterase.

It is worth noting that while these sequential inoculation strategy is inspired by conventional oenological practices, the fig matrix imparts unique selective pressures on yeast metabolism. Unlike grape must, fig juice is characterized by high sugar and pectin content, low pH, and specific amino acid profile, which may account for the observed differences in higher ester, higher alcohol, and terpenoid content compared to previous reports in grape wine. Therefore, the effectiveness of this method is not merely a transfer of technology, but an adaptation to a novel biotic environment. Future studies should employ targeted metabolomics and transcriptomics to comprehensively profile fig wine composition and the characteristics of other yeast enzymes relevant to precursor transformation, which would illuminate the fig-specific yeast interactions for releasing aroma compounds.

## 4 Conclusion

In this study, three indigenous yeast strains (*C. humilis* DBXD1, *T. delbrueckii* S14, and *S. cerevisiae* S2–7) with excellent *β*-glucosidase and esterase activity were evaluated based on their stress tolerance, volatile compound profiles, and sensory attributes in fig wine fermentation. Higher contents of esters (butanoic acid, ethyl ester, butanoic acid, methyl ester, hexanoic acid, methyl ester, ethyl acetate, isoamyl acetate, and acetic acid, hexyl ester), 3-methyl-1-butanol, nonanal, 2-nonanone, and α-terpineol (OAV > 1.0) were found in fig wine produced by DBXD1 and S14 co-inoculation prior to S2–7, which demonstrated robust tropical fruit, berries, and vanilla aromas in sensory evaluation. Valine, leucine and isoleucine degradation, glycine and serine metabolism, and glucose-alanine cycle were the main differential metabolic pathways, with acyl-CoA as the core intermediate product. Acyl-CoA subsequently involved in TCA cycle, fatty acid oxidation and degradation, amino acids metabolism, and carbohydrate metabolism, thereby improving the flavor quality of fig wines. The sequential inoculation protocol follows methods validated in grape wine fermentation, demonstrating the applicability and benefits of sequential inoculation for this underexplored substrate. Future study should perform controlled comparative fermentations across multiple fruit substrates using identical yeast strains and multiple fermentation strategies, enabling rigorous identification of fig-unique metabolic mechanism. Overall, this study provides an available solution to avoid the homogenization of flavor characteristics in fig wine, which is of great significance for the industrial production of fig wines with typical flavor and high quality.

## CRediT authorship contribution statement

**Bing Han:** Writing – original draft, Visualization, Validation, Software, Methodology, Formal analysis, Data curation. **Jian Ma:** Visualization, Software, Methodology, Investigation. **Shuai Li:** Visualization, Validation, Formal analysis. **Haihong Wu:** Visualization, Methodology, Investigation. **Yu Wang:** Visualization, Investigation, Formal analysis. **Xingye Wang:** Validation, Resources. **Yanhong Ma:** Writing – review & editing, Project administration, Conceptualization.

## Ethical statement

Sensory evaluation of this study was approved by the Human Research Ethics Committee of Jiangsu Academy of Agricultural Sciences (Authority No: PA-2025-02-005). All sensory panellists consented to participate and to have the data generated from their observations used in this publication.

## Declaration of competing interest

The authors declare that they have no known competing financial interests or personal relationships that could have appeared to influence the work reported in this paper.

## Data Availability

Data will be made available on request.

## Appendix A Supplementary data

Supplementary material: The flavor profile of fig wines with different fermentation schemes. Supplementary material
