# Supplementary material for: Aroma enhancement in fig wine through sequential fermentation with Candida humilis and Torulaspora delbrueckii: A flavoromics study
Source: Food Chem X. 2026 Apr 9;35:103855. doi: 10.1016/j.fochx.2026.103855 (PMC13092848; doi:10.1016/j.fochx.2026.103855)
Supplement: Supplementary file 1 — Supplementary material: The flavor profile of fig wines with different fermentation schemes. [file mmc1.docx]

**Table S1**

Identification and semi-quantification of volatile compound content (μg/L) in fig wines with different fermentation schemes.

|  |  | Fig wines | | | | |
| --- | --- | --- | --- | --- | --- | --- |
| No. | Volatile compounds | DV10 | S2-7 | DBXD1+S2-7 | S14+S2-7 | DBXD1+S14+S2-7 |
| 1 | Acetic acid, methyl ester | 186.62±6.09^d^ | 141.45±9.61^d^ | 413.33±14.32^c^ | 511.60±11.37^b^ | 811.84±37.75^a^ |
| 2 | Ethyl acetate | 24344.68±  1334.25^c^ | 23136.34±  1381.69^c^ | 35238.04±  1008.56^b^ | 36429.38±  1114.97^b^ | 49845.49±  1397.25^a^ |
| 3 | n-Propyl acetate | N.D. | N.D. | N.D. | N.D. | 2246.19±138.68^a^ |
| 4 | Acetic acid, butyl ester | 356.23±24.44^a^ | 134.03±15.54^c^ | 369.78±26.29^a^ | 231.77±24.93^b^ | 362.60±26.09^a^ |
| 5 | Isobutyl acetate | 728.38±31.59^c^ | 289.85±11.91^e^ | 915.22±93.95^b^ | 517.72±29.63^d^ | 1248.81±75.58^a^ |
| 6 | Isoamyl acetate | 5525.74±  366.02^c^ | 6505.64±  359.46^c^ | 28511.36±  1318.02^b^ | 21903.87±  895.68^b^ | 49017.46±  1269.12^a^ |
| 7 | Acetic acid, hexyl ester | N.D. | N.D. | N.D. | N.D. | 221.82±10.57^a^ |
| 8 | Acetic acid, phenylmethyl ester | 282.90±16.63^b^ | 444.17±18.64^a^ | N.D. | N.D. | N.D. |
| 9 | Acetic acid, 2-phenylethyl ester | 1347.96±140.52^b^ | 1763.47±107.81^b^ | 4986.04±279.49^a^ | 4418.71±222.10^a^ | 4788.67±262.13^a^ |
| 10 | Butanoic acid, ethyl ester | 1478.77±102.83^d^ | 1006.57±89.23^d^ | 6395.48±242.20^b^ | 5542.73±129.35^cc^ | 8613.78±308.16^a^ |
| 11 | Pentanoic acid, ethyl ester | 271.70±18.86^a^ | 127.79±15.41^b^ | N.D. | 220.56±26.45^a^ | N.D. |
| 12 | Hexanoic acid, ethyl ester | 10394.46±  559.40^c^ | 14270.97±  1092.41^c^ | 53910.22±  2470.60^a^ | 56562.53±  2589.07^a^ | 43534.35±  1660.63^b^ |
| 13 | Heptanoic acid, ethyl ester | 187.22±24.64^b^ | 140.74±15.13^c^ | 226.02±11.44^a^ | 248.02±17.52^a^ | 241.23±15.39^a^ |
| 14 | Octanoic acid, ethyl ester | 25223.60±  1530.42^c^ | 27559.87±  1598.83^c^ | 98788.16±  5767.15^a^ | 97421.03±  3584.32^a^ | 73829.93±  3896.46^b^ |
| 15 | Nonanoic acid, ethyl ester | N.D. | N.D. | N.D. | 4946.65±497.81^a^ | N.D. |
| 16 | Decanoic acid, ethyl ester | 33521.49±  543.24^d^ | 56248.47±  1189.98^c^ | 84574.44±  1698.94^b^ | 93036.53±  2848.27^a^ | 54053.24±  3248.34^c^ |
| 17 | Ethyl 9-decenoate | 7539.90±  574.30^e^ | 11473.38±  892.57^d^ | 22522.15±  1511.44^a^ | 18707.99±  693.00^b^ | 15159.33±  1073.94^c^ |
| 18 | Dodecanoic acid, ethyl ester | 7878.50±  274.55^e^ | 13420.29±  998.13^c^ | 15042.93±  1256.81^a^ | 14045.43±  856.66^b^ | 11395.81±  574.21^d^ |
| 19 | Tetradecanoic acid, ethyl ester | 5114.17±288.75^a^ | 4713.75±327.41^b^ | 1649.14±115.75^c^ | 1640.49±132.10^c^ | 449.45±31.66^d^ |
| 20 | Hexadecanoic acid, ethyl ester | 7398.29±327.04^b^ | 7081.09±320.40^c^ | 7843.49±361.46^a^ | 7135.25±219.54^c^ | 4881.50±310.17^d^ |
| 21 | Benzoic acid, ethyl ester | 735.65±31.68^c^ | 891.23±64.24^b^ | 700.92±44.34^c^ | 1012.02±41.66 ^a^ | 969.85±84.08^a^ |
| 22 | Propanoic acid, 2-hydroxy-, ethyl ester, (S)- | 312.24±16.55^b^ | 409.89±55.03^a^ | N.D. | 127.80±11.46^c^ | N.D. |
| 23 | Formic acid, octyl ester | 410.92±32.23^d^ | 440.49±28.71^d^ | 777.15±24.82^c^ | 986.71±34.74^b^ | 1223.69±80.02^a^ |
| 24 | Butanoic acid, methyl ester | 1488.26± 197.90^c^ | 1193.32±155.69^d^ | 1668.29±136.92^b^ | 1414.42±124.53^c^ | 1833.15±122.78^a^ |
| 25 | Hexanoic acid, methyl ester | 1030.69±72.75^b^ | 941.11±50.74^b^ | 479.21±30.14^c^ | 1292.70±58.54^a^ | 1353.09±95.32^a^ |
| 26 | Isopentyl hexanoate | N.D. | N.D. | 838.76±46.00^b^ | 1185.91±89.74^a^ | N.D. |
| 27 | Heptanoic acid, methyl ester | 912.69±42.73^d^ | 1346.25±79.79^c^ | 2207.14±116.63^a^ | 2322.89±131.26^a^ | 1643.98±175.07^b^ |
| 28 | Octanoic acid, methyl ester | 978.86±52.89^d^ | 1006.97±72.26^d^ | 2743.78±112.11^b^ | 2955.70±134.39^a^ | 2345.55±105.89^c^ |
| 29 | Octanoic acid, 3-methylbutyl ester | N.D. | 346.83±20.58^c^ | 2149.82±145.66^a^ | 2135.39±100.90^a^ | 1856.18±132.05^b^ |
| 30 | Nonanoic acid, methyl ester | 296.36±12.06^d^ | 317.14±24.25^c^ | 348.98±17.55^b^ | 928.94±28.97^a^ | 298.95±15.59^d^ |
| 31 | Decanoic acid, methyl ester | 1729.93±125.60^d^ | 3622.80±78.46^c^ | 5914.82±124.14^b^ | 6854.40±267.33^a^ | 3707.00±169.13^c^ |
| 32 | Pentadecanoic acid, 3-methylbutyl ester | 201.12±15.58^d^ | 405.37±26.81^c^ | 645.55±28.73^b^ | 732.27±20.34^a^ | 657.32±14.53^b^ |
| 33 | Dodecanoic acid, methyl ester | 953.98±43.76^b^ | 1217.68±96.23^a^ | 996.41±31.90^b^ | 807.47±27.01^c^ | 662.31±34.35^d^ |
| 34 | Methyl tetradecanoate | 397.10±33.05^a^ | 394.66±23.03^a^ | N.D. | N.D. | N.D. |
| 35 | Hexadecanoic acid, methyl ester | 990.53±31.59^d^ | 985.43±73.81^d^ | 1204.89±62.26^b^ | 1457.02±48.88^a^ | 1123.93±49.10^c^ |
|  | Total esters | 142218.96±  12255.55^d^ | 181977.05±  25265.52^c^ | 382061.52±  24680.32^a^ | 387733.88±  19479.06^a^ | 338376.45±  15432.92^b^ |
| 36 | 1-Propanol, 2-methyl- | 4954.90±106.95^c^ | 4069.41±128.57^d^ | 6464.34±285.08^b^ | 5084.96±191.22^c^ | 8252.24±225.45^a^ |
| 37 | 1-Butanol | 889.20±34.50^b^ | 450.94±31.80^d^ | 935.67±53.81^a^ | 417.05±32.29^d^ | 528.59±29.21^c^ |
| 38 | 1-Butanol, 3-methyl- | 90982.89±  3140.86^c^ | 84981.42±  2574.50^d^ | 129049.62±  6358.32^b^ | 124798.81±  5817.38^b^ | 148413.36±  4748.85^a^ |
| 39 | 2,3-Butanediol | 2367.87±167.43^d^ | 1033.25±73.06^e^ | 2819.12±192.70^b^ | 3475.64±188.54^a^ | 2634.05±162.48^c^ |
| 40 | 2,3-Butanediol, [R-(R*,R*)]- | 2139.65±101.77^a^ | 1192.97±73.34^c^ | 1254.60±63.58^c^ | 1731.99±49.92^b^ | 675.61±43.37^d^ |
| 41 | 2,4-Pentanediol | N.D. | 700.20±38.13^c^ | 848.04±69.12^b^ | N.D. | 1028.02±71.17^a^ |
| 42 | 1-Hexanol | 475.57±27.44^c^ | 487.57±29.03^c^ | 512.51±32.03^b^ | 596.41±35.42^a^ | 514.06±36.78^b^ |
| 43 | 2-Hexanol, 3-methyl- | 551.10±40.21^b^ | 857.35±62.71^a^ | 381.23±23.23^d^ | 448.38±26.09^c^ | 341.06±29.96^d^ |
| 44 | 2-Hexanol, 5-methyl- | 722.76±46.98^b^ | 627.88±32.23^c^ | 761.85±26.20^b^ | 737.21±30.18^b^ | 1135.53±31.03^a^ |
| 45 | 2-Heptanol, 6-methyl- | 501.13±27.34^c^ | 331.18±16.49^d^ | 652.07±23.81^a^ | 208.04±10.70^e^ | 599.80±24.50^b^ |
| 46 | 2-Nonanol | 177.02±10.52^e^ | 358.04±16.49^d^ | 1003.25±20.11^c^ | 1864.23±81.93^a^ | 1649.68±114.38^b^ |
| 47 | 1-Decanol | 139.68±15.39^e^ | 212.83±26.73^d^ | 435.70±15.28^b^ | 349.79±20.67^c^ | 536.61±38.77^a^ |
| 48 | Benzyl alcohol | 19017.83±  985.14^a^ | 20412.78±  2508.83^a^ | 1935.01±  114.06^c^ | 3893.78±  1228.72^b^ | 1471.31±  107.15^d^ |
| 49 | Phenylethyl alcohol | 32091.84±  2151.30^c^ | 37696.33±  2004.23^b^ | 60704.87±  2240.11^a^ | 61696.02±  2312.47^a^ | 46533.09±  1220.09^b^ |
|  | Total alcohols | 155011.45±  14207.80^b^ | 153412.15±  24160.58^b^ | 207757.88±  17506.83^a^ | 205302.29±  14732.89^a^ | 214313.01±  16127.04^a^ |
| 50 | Nonanal | 511.35±20.65^d^ | 411.05±28.76^e^ | 945.69±39.87^b^ | 836.95±32.14^c^ | 2046.13±179.77^a^ |
| 51 | Tetradecanal | 592.92±25.31^c^ | 554.00±26.45^c^ | 948.95±38.97^a^ | 910.88±65.94^a^ | 766.58±43.22^b^ |
| 52 | 2-Nonanone | 112.40±8.35^e^ | 278.73±17.09^d^ | 1280.02±88.78^b^ | 638.52±38.31^c^ | 2059.67±156.98^a^ |
|  | Total aldehydes and ketones | 1216.67±75.97^e^ | 1243.78±81.88^e^ | 3174.66±177.54^b^ | 2386.36±136.39^c^ | 4872.38±147.59^a^ |
| 53 | Acetic acid | 16199.65±  808.53^b^ | 24023.57±  1196.37^a^ | 9110.04±  578.09^d^ | 14390.89±  1371.59^c^ | 8221.31±  372.76^e^ |
| 54 | Propanoic acid, 2-methyl- | 184.07±12.20^b^ | 161.62±19.98^b^ | 134.76±14.65^c^ | 173.16±12.52^b^ | 360.06±28.84^a^ |
| 55 | Octanoic acid | 4249.54±  385.45^d^ | 5393.39±  370.67^c^ | 27524.25±  1763.33^b^ | 26411.34±  1745.50^b^ | 32743.05±  1501.74^a^ |
| 56 | Nonanoic acid | N.D. | N.D. | N.D. | 5174.41±265.86^a^ | 4071.43±287.93^b^ |
| 57 | n-Decanoic acid | 2171.91±179.14^e^ | 2639.46±193.06^d^ | 5256.31±298.97^c^ | 6674.71±295.39^b^ | 9244.26±487.78^a^ |
|  | Total acids | 22805.17±  1214.42^e^ | 32218.05±  2327.12^d^ | 42025.36±  2107.80^c^ | 52824.51±  2784.72^b^ | 54640.10±  2001.94^a^ |
| 58 | β-Myrcene | 733.92±56.31^a^ | 593.30±32.14^d^ | 628.55±43.86^c^ | 486.46±28.20^e^ | 667.82±31.97^b^ |
| 59 | D-Limonene | 290.83±19.06^a^ | 254.13±17.70^b^ | N.D. | 291.61±23.25^a^ | 255.81±18.88^b^ |
| 60 | β-Ocimene | 913.96±49.43^c^ | 983.51±56.37^b^ | N.D. | 1299.37±118.79^a^ | N.D. |
| 61 | Rose oxide | 169.64±19.25^a^ | 173.63±16.36^a^ | N.D. | N.D. | N.D. |
| 62 | Nerol oxide | 1275.26±111.32^d^ | 1342.35±145.98^c^ | 1134.34±150.32^e^ | 1979.35±195.10^a^ | 1677.80±163.56^b^ |
| 63 | 3-Hexen-1-ol, (Z)- | 92.86±11.48^b^ | 130.18±18.39^a^ | N.D. | N.D. | N.D. |
| 64 | Linalool | 25652.55±  1060.62^a^ | 26722.59±  1682.28^a^ | 20769.28±  1628.79^c^ | 26018.49±  1309.30^a^ | 22076.35±  1214.73^b^ |
| 65 | 1,5,7-Octatrien-3-ol, 3,7-dimethyl- | 1465.74±166.99^c^ | 1526.84±202.72^b^ | 1224.05±140.18^e^ | 1635.43±106.98^a^ | 1326.25±143.52^d^ |
| 66 | α-Terpineol | 3009.42±167.31^e^ | 3172.13±198.59^d^ | 3557.72±115.12^c^ | 3628.83±185.58^b^ | 3714.22±101.45^a^ |
| 67 | Citronellol | 2507.15±173.72^b^ | 2964.44±298.59^a^ | 2199.45±171.74^c^ | 2098.41±247.35^a^ | 1663.92±120.16^d^ |
| 68 | β-Damascenone | 258.45±12.11^b^ | 317.70±22.82^a^ | N.D. | N.D. | N.D. |
|  | Total terpenoids | 36369.79±  1284.22^b^ | 38180.80±  1425.41^a^ | 29513.40±  1839.72^d^ | 37437.96±  1443.27^ab^ | 31382.17±  1610.62^c^ |

Note: The serial numbers of No. column were consistent with the codes of volatile compounds in Fig. 3C and Fig. 3H, respectively. Different letters (a-e) in the same row indicated significant differences in volatile compounds among different groups (*P* < 0.05). N.D.: not detected or below the quantitation limit.

**Table S2** The odor activity values (OAVs) in fig wines with different fermentation schemes.

|  |  |  | Fig wines | | | | |
| --- | --- | --- | --- | --- | --- | --- | --- |
| Volatile compounds | Threshold  (µg/L) | Odor descriptors | DV10 | S2-7 | DBXD1+S2-7 | S14+S2-7 | DBXD1+S14+S2-7 |
| Ethyl acetate | 7500 | Apple, pineapple, floral | 3.25 | 3.08 | 4.70 | 4.86 | 6.65 |
| Isoamyl acetate | 30 | Banana, fruity, sweet | 184.19 | 216.85 | 950.38 | 730.13 | 1633.92 |
| Acetic acid, hexyl ester | 45 | Pear, fruity, sweet | N.D. | N.D. | N.D. | N.D. | 4.93 |
| Acetic acid, 2-phenylethyl ester | 250 | Flowery, rose, honey | 5.39 | 7.05 | 19.94 | 17.67 | 19.15 |
| Butanoic acid, ethyl ester | 20 | Strawberry, pineapple, apple | 73.94 | 50.33 | 319.77 | 277.14 | 430.69 |
| Hexanoic acid, ethyl ester | 14 | Green apple, pineapple | 742.46 | 1019.35 | 3850.73 | 4040.18 | 3109.60 |
| Heptanoic acid, ethyl ester | 220 | Fruity, fatty, pineapple | 0.85 | 0.64 | 1.03 | 1.13 | 1.10 |
| Octanoic acid, ethyl ester | 580 | Pineapple, pear, floral | 43.49 | 47.52 | 170.32 | 167.97 | 127.29 |
| Decanoic acid, ethyl ester | 200 | Grape, apple, fruity | 167.61 | 281.24 | 422.87 | 465.18 | 270.27 |
| Ethyl 9-decenoate | 100 | Fruity, waxy, cheese | 75.40 | 114.73 | 225.22 | 187.08 | 151.59 |
| Dodecanoic acid, ethyl ester | 500 | Sweet, floral, fruity | 15.76 | 26.84 | 30.09 | 28.09 | 22.79 |
| Tetradecanoic acid, ethyl ester | 2000 | Mild, waxy, soapy | 2.56 | 2.36 | 0.82 | 0.82 | 0.22 |
| Hexadecanoic acid, ethyl ester | 1000 | Fatty, fruity, sweet | 7.40 | 7.08 | 7.84 | 7.14 | 4.88 |
| Benzoic acid, ethyl ester | 60 | Wintergreen, fruity | 12.26 | 14.85 | 11.68 | 16.87 | 16.16 |
| Butanoic acid, methyl ester | 7.1 | Fruity, apple | 209.61 | 168.07 | 234.97 | 199.21 | 258.19 |
| Hexanoic acid, methyl ester | 70 | Fruity, pineapple | 14.72 | 13.44 | 6.85 | 18.47 | 19.33 |
| Octanoic acid, methyl ester | 220 | Intense citrus, rose | 4.45 | 4.58 | 12.47 | 13.44 | 10.66 |
| Octanoic acid, 3-methylbutyl ester | 125 | Sweet, fruity, floral | N.D. | 2.77 | 17.20 | 17.08 | 14.85 |
| Decanoic acid, methyl ester | 1200 | Waxy, fruity | 1.44 | 3.02 | 4.93 | 5.71 | 3.09 |
| 1-Butanol, 3-methyl- | 30000 | Banana, mellow, whiskey | 3.03 | 2.83 | 4.30 | 4.16 | 4.95 |
| 2-Hexanol, 3-methyl- | 560 | Fruity | 0.98 | 1.53 | 0.68 | 0.80 | 0.61 |
| 2-Nonanol | 75 | Citrus, cheese | 2.36 | 4.77 | 13.38 | 24.86 | 22.00 |
| Benzyl alcohol | 200 | Almonds | 95.09 | 102.06 | 9.68 | 19.47 | 7.36 |
| Phenylethyl alcohol | 14000 | Rose, honey | 2.29 | 2.69 | 4.34 | 4.41 | 3.32 |
| Nonanal | 15 | Rose, orris, citrus | 34.09 | 27.40 | 63.05 | 55.80 | 136.41 |
| 2-Nonanone | 41 | Fruity, floral, herbal, fatty | 2.74 | 6.80 | 31.22 | 15.57 | 50.24 |
| Acetic acid | 200000 | Vinegar | 0.08 | 0.12 | 0.05 | 0.07 | 0.04 |
| Octanoic acid | 8800 | Fatty, waxy, cheese | 0.48 | 0.61 | 3.13 | 3.00 | 3.72 |
| β-Myrcene | 670 | Fatty, Sweet | 1.10 | 0.89 | 0.94 | 0.73 | 1.00 |
| Linalool | 15 | Rose, citrus, lavender, lemony | 1710.17 | 1781.51 | 1384.62 | 1734.57 | 1471.76 |
| α-Terpineol | 250 | Lilac, citrus | 12.04 | 12.69 | 14.23 | 14.52 | 14.86 |
| Citronellol | 100 | Rose, citrus | 25.07 | 29.64 | 21.99 | 20.98 | 16.64 |
| Rose oxide | 0.2 | Floral, green, rose | 848.19 | 868.13 | N.D. | N.D. | N.D. |
| β-Damascenone | 0.05 | Baked apple-like, grape juice-like | 5168.92 | 6354.05 | N.D. | N.D. | N.D. |

N.D.: not detected or below limit of quantitation.
